# Predictors of lymphovascular invasion identified from pathological factors in Chinese patients with breast cancer

**DOI:** 10.18632/oncotarget.23503

**Published:** 2017-12-20

**Authors:** Sandi Shen, Gaofang Xiao, Richang Du, Ningdong Hu, Xu Xia, Haibo Zhou

**Affiliations:** ^1^ Thoracic Surgery, The Sixth Affiliated Hospital of Guangzhou Medical University, Qingyuan People's Hospital, Qingyuan, P. R. China; ^2^ Department of Pathology, Yuebei People's Hospital, Shantou University, Shaoguan, P. R. China

**Keywords:** invasive breast cancer, lymphovascular invasion, predictor, pathological factor

## Abstract

This study aimed to evaluate correlations between lymphovascular invasion (LVI) and the expression of estrogen receptor (ER), progesterone receptor (PR), human epidermal growth factor receptor-2 (HER-2), Ki-67, CK5/6, epidermal growth factor receptor (EGFR), vascular endothelial growth factor (VEGF), E-cadherin, BCL11A and P53 in invasive breast cancer and to identify predictors of LVI based on these pathological factors. In all, 392 paraffin-embedded tissues from consecutive patients with primary operable invasive breast cancer were included. Immunohistochemistry (IHC) was retrospectively performed using a tissue microarray (TMA) of the paraffin-embedded tissues. LVI-positive rates were compared using the χ^2^ test. Correlations between pathological factors were assessed using Spearman's test. Binary logistic regression was employed in multivariate analyses of statistically significant factors. The results showed that LVI positivity was significantly higher in patients with HER-2-positive expression or high Ki-67 expression. HER-2 expression was weakly positively correlated with Ki-67 expression. HER-2-positive expression and high Ki-67 expression were found to be risk factors for LVI, and associations between LVI and other pathological factors were not significant. Therefore, HER-2-positive expression and high Ki-67 expression are predictors of LVI, whereas the expression of ER, PR, CK5/6, EGFR, VEGF, E-cadherin, BCL11A and P53 is not associated with LVI in invasive breast cancer.

## INTRODUCTION

The prognosis of breast cancer is determined by certain clinicopathological factors, including the presence or absence of lymph node metastasis, tumor size, histological grade, estrogen receptor (ER) status, progesterone receptor (PR) status, human epidermal growth factor receptor-2 (HER-2) status, Ki-67 expression, and the presence or absence of lymphovascular invasion (LVI) [1]. LVI is the main route of lymph node metastasis and is a known independent predictor of lymph node metastasis, disease-free survival (DFS) and overall survival (OS) in breast cancer [2]. However, the mechanisms that underlie the development of LVI remain controversial. Since LVI presents in peritumoral vessels, the canonical view is that cancer cells invade lymphatic vessels and blood vessels. Conversely, Sepi Mahooti reported that LVI can result from encircling lymphovasculogenesis [3].

The BCL11A gene was first discovered in mice as a site for retroviral insertion and was initially referred to as CTIP1, also known as EVI9 [4]. It was later found in the human chromosome 2p16.1 region, has a total length of approximately 102 kb and encodes a Kruppel-like zinc finger protein [5]. The N-terminus of the protein has a constant C2HC zinc finger, and the C-terminus contains six C2HC zinc finger structures. Previous studies have shown that BCL11A binds to the 5′-GGCCGG-3′ motif of the promoter region of a target gene and affects the deacetylation of histone H3/H4, thereby inhibiting transcription of the target gene [6]. BCL11A is also a novel breast cancer gene that has been significantly correlated with a high histological grade; moreover, it is overexpressed in triple-negative breast cancer (TNBC) [7]. No studies have reported the relationships between LVI and BCL11A expression, and studies on the associations between LVI and the expression of ER, PR, HER-2, Ki-67, CK5/6, epidermal growth factor receptor (EGFR), vascular endothelial growth factor (VEGF), E-cadherin and P53 in invasive breast cancer have produced controversial results. Therefore, the current study aimed to investigate correlations between LVI and these pathological factors and to identify predictors of LVI from among these factors.

## RESULTS

### Comparison of LVI-positive rates in relation to different pathological factors

In all, 392 female patients with invasive breast cancer were included in this analysis. The LVI-positive rate was significantly high in patients whose tumors were positive for HER-2 expression (χ^2^=20.233, *P*<0.001) and high Ki-67 expression (χ^2^=10.230, *P*=0.001). LVI-positive rates did not show significant associations with the expression of ER, PR, CK5/6, EGFR, VEGF, E-cadherin, BCL11A or P53 (Table 1).

**Table 1:** Association between LVI and other pathological factors in invasive breast cancer

Pathological factors	No. with LVI		Positive rate	χ^2^	*P*
	Positive	Negative			
ER expression					
Positive	65	197	24.8%	1.127	0.288
Negative	26	104	20.0%		
PR expression					
Positive	48	185	20.6%	2.201	0.138
Negative	43	116	27%		
HER-2 expression					
Positive	37	54	40.7%	20.233	0.001
Negative	54	247	17.9%		
Ki-67 expression					
High	57	131	30.3%	10.230	0.001
Low	34	170	16.7%		
CK5/6 expression					
Positive	20	76	20.8%	0.404	0.525
Negative	71	225	24%		
EGFR expression					
Positive	20	60	25.0%	0.18	0.672
Negative	71	241	22.8%		
VEGF expression					
Positive	36	103	25.9%	0.871	0.351
Negative	55	198	21.7%		
E-cadherin expression					
Positive	59	203	22.5%	0.214	0.643
Negative	32	98	24.6%		
BCL11A expression					
Positive	35	92	27.6%	1.989	0.158
Negative	56	209	21.1%		
P53 expression					
Positive	45	119	27.4%	2.823	0.093
Negative	46	182	20.2%		

**Figure 1:**
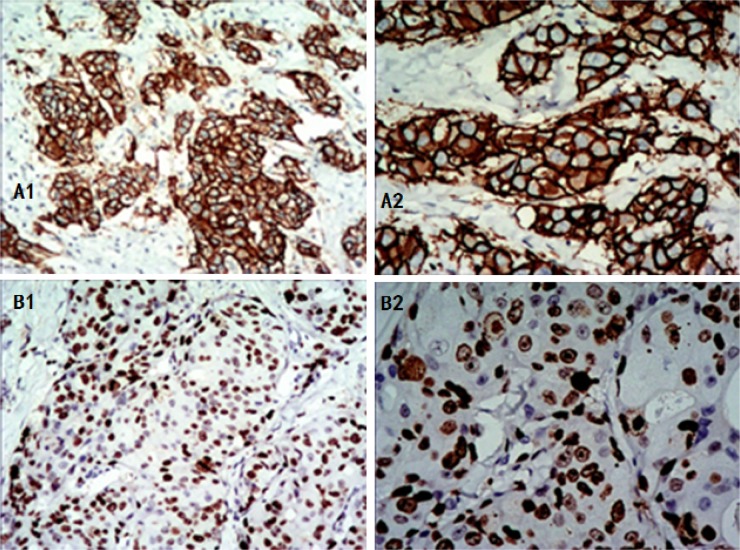
Positive expression of HER-2 and Ki-67 in invasive breast cancer as shown by IHC Positive HER-2 expression: the cell membrane is brown and continuous. **(A1)** 100x magnification, **(A2)** 200x magnification. High Ki-67 expression: the nucleus is brown, with tumor cell positivity in 70% of the cells, **(B1)** 100x magnification, **(B2)** 200x magnification. IHC: immunohistochemistry.

Immunohistochemistry (IHC) images for HER-2 and Ki-67 are shown in Figure 1.

### Correlations among HER-2, Ki-67 and pathological factors

HER-2 expression was weakly positively correlated with Ki-67 expression (CC=0.234, *P*<0.001) and P53 expression (CC=0.128, *P*=0.011) and weakly negatively correlated with CK5/6 (CC=-0.116, *P*<0.05), ER (CC=-0.280, *P*<0.001) and PR (CC=-0.309, *P*<0.001) expression. Ki-67 expression was weakly positively correlated with EGFR (CC=0.198, *P*<0.001), VEGF (CC=0.110, *P*=0.029) and P53 (CC=0.128, *P*=0.011) expression and weakly negatively correlated with ER (CC=-0.257, *P*<0.001) and PR (CC=-0.195, *P*<0.001) expression. No relationships were observed for the other pathological factors (Table 2).

**Table 2:** Correlation coefficients among HER-2, Ki-67 and pathological factors in invasive breast cancer

Pathological factors	Correlation coefficients								
	Ki-67	CK5/6	EGFR	VEGF	E-cadherin	BCL11A	P53	ER	PR
HER-2	0.234^**^	−0.116^*^	−0.054	−0.016	−0.011	0.007	0.134^**^	−0.280^**^	−0.309^**^
Ki-67	−	0.023	0.198^**^	0.110^*^	−0.007	0.001	0.128^*^	−0.257^**^	−0.195^**^

The data were analyzed using Spearman's test. ^*^ indicates *P*<0.05 (two-tailed); ^**^ indicates *P*<0.01 (two-tailed).

### Multivariate logistic regression analyses of pathological factors associated with LVI

Based on a multivariate analysis, HER-2-positive expression was found to be a strong risk factor for LVI (odds ratio (OR)=2.719, confidence interval (CI): 1.606-4.604, *P*<0.001). Ki-67 expression was also found to be a risk factor for LVI (OR=1.785, CI: 1.081-2.947, *P*=0.024) (Table 3).

**Table 3:** Logistic regression analysis of LVI with pathological factors in invasive breast cancer

Pathological factors	Multivariate	
	OR (95% CI)	*P*
HER-2 expression		
positive vs. negative	2.719 (1.606-4.604)	<0.001
Ki-67 expression		
high vs. low	1.785 (1.081-2.947)	0.024

## DISCUSSION

HER-2 is a tyrosine kinase receptor that activates critical signal transduction pathways. HER-2-positive expression can promote cell migration and invasion [8], resulting in highly aggressive breast cancer with poor outcomes. HER-2 positivity occurs in 15–20% of all breast cancers and is associated with increased metastatic potential and poor patient survival. Moreover, HER-2-targeted therapies can improve the prognosis of patients whose tumors are positive for HER-2 expression [9, 10]. In the current study, patients with HER-2-positive expression showed significantly high positive rates for LVI. Tan et al. also reported that HER-2 positivity is associated with LVI [11]. Multivariate analyses showed that HER-2-positive expression is a strong independent risk factor for LVI (OR=2.719), which is consistent with previous reports [12].

Ki-67 expression is a predictor of breast cancer and has been identified as an independent prognostic factor of DFS in invasive breast cancer [13]. High Ki-67 expression is the result of rapid tumor proliferation, which results in a poor prognosis [14]. In the current study, patients with high Ki-67 expression had significantly high rates of LVI compared to those with low Ki-67 expression. In addition, a multivariate analysis showed that high Ki-67 expression is a risk factor for LVI (OR=1.785). However, Mohamed et al. reported that Ki-67 expression was not associated with LVI [14]. Two primary reasons likely explain this difference: First, the races and regions of the patients were different; second, the criterion of high Ki-67 expression was different. The cut-off value we used was ≥14% according to the St Gallen International Expert Consensus [15], while the cut-off value adopted by Mohamed et al. was ≥25%. HER-2 expression is positively correlated with Ki-67 and P53 expression, and Ki-67 expression is positively correlated with EGFR, VEGF and P53 expression. In this study, the patients had P53 mutations, and with the loss of apoptosis induction, P53 promotes malignant transformation. Positive HER-2, EGFR and VEGF expression can promote tumor growth. Therefore, the observed relationships among the pathological factors indicate that the rapid growth of tumor cells should be an important factor for LVI.

In the current study, no significant differences were found in the rates of LVI between tumors with positive BCL11A expression and those with negative BCL11A expression. To the best of our knowledge, this is the first report of such relationships. BCL11A is overexpressed in TNBC [7], and LVI is less frequent in TNBC than in other subtypes [12]. Therefore, BCL11A may serve as a good biomarker for the identification of TNBC, without affecting LVI [16].

In the current study, EGFR and VEGF expression were not correlated with LVI, which did not support the hypothesis that LVI can result from encircling lymphovasculogenesis. LVI also showed no correlations with ER, PR, CK5/6, E-cadherin or P53 expression. Therefore, we inferred that LVI was the result of rapid tumor growth.

In summary, the results of this study show that positive HER-2 expression and high Ki-67 expression are associated with LVI and are predictors of LVI in invasive breast cancer.

## MATERIALS AND METHODS

In all, 392 paraffin-embedded tissue samples were collected from consecutive patients with primary operable invasive breast cancer who were diagnosed between 2010 and 2015 at Yuebei People's Hospital. All data were anonymized prior to analysis. This study was approved by the Institutional Review Board of Guangzhou Medical University. None of the enrolled patients underwent radiotherapy, chemotherapy or hormone therapy prior to the extraction of pathological specimens. The samples were collected from lumpectomy or mastectomy specimens, fixed in 10% neutral buffered formalin, and embedded in paraffin. To analyze pathological factors, immunohistochemical staining was retrospectively performed on the collected tissues, which were arranged in a microarray (24 tissue cores per block, 0.6 mm in diameter). The donor tissue blocks containing the most representative and well-preserved tumor areas were marked by a pathologist according to the corresponding hematoxylin & eosin (H&E)-stained slides. These blocks were screened for immunohistochemical staining. Each sample was obtained from the corresponding case and mounted in a recipient block. The following pathological factors were retrospectively analyzed: the expression of ER, PR, HER-2, Ki-67, CK5/6, EGFR, VEGF, E-cadherin, BCL11A and P53 and the presence of LVI.

### Definition of LVI

**Figure 2:**
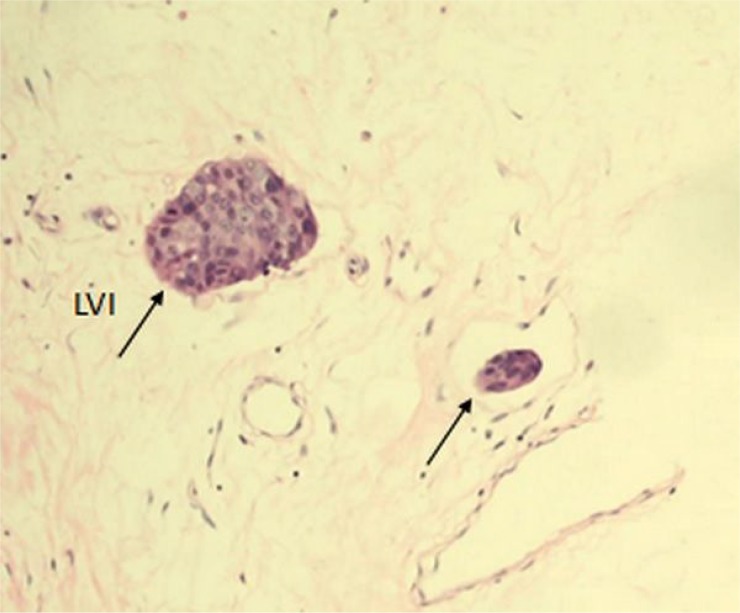
Tumor LVI (single arrows) in a section of invasive breast cancer stained with H&E at 200x magnification LVI: lymphovascular invasion.

LVI was assessed on H&E-stained sections of original cancer tissues. LVI was defined as the presence of cancer cells within a definite endothelial-lined space (lymphatic or blood vessel) (Figure 2). LVI included both lymphatic and blood vessel invasion.

### Immunohistochemistry

Each tissue microarray block was cut into 2.5-μm-thick consecutive sections that were routinely stained with monoclonal antibodies against BCL11A (ab19487) (Abcam, Inc., Cambridge, MA); dilution 1:250), ER (ZM-0104), PR (ZM-0215), HER-2 (ZM-0065), Ki-67 (ZM-0167), CK5/6 (ZM-0313), EGFR (ZM-0093), VEGF (ZM-0625), E-cadherin (ZM-0092) and P53 (ZM-0408). The last nine antibodies were ready-to-use and purchased from OriGene (Beijing, CHN). A positive control was obtained from a breast cancer tissue sample with positive immunohistochemical staining results, and a negative control was taken from a paraffin-embedded breast cancer tissue sample that was not incubated with antibodies. The tissue sections were deparaffinized in TO (a substitute for xylene) and then rehydrated through decreasing concentrations of ethanol. Peroxidase activity was inhibited by incubating the sections in 3% H_2_O_2_ for 10 minutes at room temperature. The sections were heated in citrate buffer (pH 9.0) in a high-pressure cooker for approximately 3.5 minutes (counting began at jet formation) for antigen retrieval and then incubated with the corresponding primary antibodies at 37°C for 1.5 hours. The sections were washed and incubated with a secondary antibody (OriGene; PV-6000) at room temperature for 30 minutes. Subsequently, the sections were treated with 3,3’-diaminobenzidine (DAB) (OriGene; ZLI-9019; dilution 1:30) for 3-5 minutes. Then, the sections were counterstained, dehydrated and mounted with DPX mounting medium. Immunohistochemical staining was evaluated by a pathologist using a light microscope (Olympus BX53, Japan). The cut-off value for ER and PR was ≥1% positive nuclear staining in the tumor. HER-2 status was assessed using IHC as follows: scores of 0 and 1+ were regarded as negative; 2+ was regarded as equivocal, warranting a further test using in situ hybridization (ISH); and 3+ was regarded as positive. The cut-off value used to indicate high Ki-67 nuclear expression was ≥14%. The cut-off value for positive CK5/6 expression was ≥10% cytoplasmic staining. The cut-off value for positive EGFR expression was ≥10% cell membrane staining. The cut-off value for positive VEGF expression was ≥25% cytoplasmic staining. The cut-off value for positive E-cadherin expression was ≥50% cell membrane staining. The cut-off value for positive BCL11A expression was ≥10% and moderate or strong nuclear or cytoplasmic staining. The cut-off value for positive P53 expression was ≥10% nuclear staining.

### Statistical analysis

Statistical analysis was performed using SPSS version 20.0 (SPSS Inc., Chicago, IL, USA). LVI-positive rates were compared across different pathological factors using the χ^2^ test. Correlations between pathological factors were assessed using Spearman's test. To predict LVI, binary logistic regression using the “enter” method was used for statistically significant factors in multivariate analyses. All statistical analyses were 2-sided, with significance defined as a *P* value <0.05. The OR and 95% CI were calculated for each variable.
